# Glass Transition and Related Phenomena

**DOI:** 10.3390/ijms24108685

**Published:** 2023-05-12

**Authors:** Andrzej Grzybowski

**Affiliations:** Institute of Physics, University of Silesia in Katowice, ul. 75 Pułku Piechoty 1, 41-500 Chorzów, Poland; andrzej.grzybowski@us.edu.pl

Despite recent advances in the study of complex systems, which were recognized by the Nobel Prize in Physics in 2021, glass transition and the physicochemical phenomena that occur in the supercooled liquid and glassy states have remained shrouded, at least partially, in mystery for various material groups. For this reason, the Special Issue entitled “Glass Transition and Related Phenomena” was developed and constitutes a broad platform, giving opportunities to present results of experimental, simulation, and theoretical investigations and various ideas to gain a deeper insight into understanding the thermodynamically metastable and nonequilibrium states in the vicinity of the liquid–glass transition. The scientific reports published in this Special Issue cover various topics investigated using experimental and simulation techniques and represent progress toward the development of a consistent viewpoint on the cognitive and application aspects considered for non-crystalline states of condensed matter.

Papers [1,2,3,4], based on the results of computer simulations, constitute an interesting collection of successful applications of isotropic and anisotropic models in equilibrium and non-equilibrium molecular dynamics (MD) computations, which are even extended to the study of dynamic properties using artificial intelligence methods [4] that become unavoidably necessary research tools. Lagogianni and Varnik [1] studied metallic glasses and their deformations, called shear bands, that hamper the application of such glasses as structural components. They performed non-equilibrium molecular dynamics simulations in the Kob-Andersen binary Lennard-Jones mixture (KABLJ), which is a commonly known an isotropic model consisting of two point species interacting via the LJ forces. Since the last decade of the 20th century, the KABLJ model has been intensively used to simulate the physicochemical properties of supercooled liquids and glasses, and it is still very useful in many cases, including metallic glasses, for which Lagogianni and Varnik show, among other things, that the temperature inside the sample first remains essentially constant in the early elastic deformation regime but then starts to rise as soon as plastic deformation begins. Such a heat generation may cause serious consequences for the mechanical properties of metallic glasses, which indicates the need for further investigations in this field.

Despite many successful applications of the KABLJ model, including [1], we should not refrain from pointing out that the KABLJ model has deficiencies consisting in its isotropic character that favors its computational efficiency but fails to properly reflect anisotropies of molecular shapes and interactions, which dominate in real molecular liquids. A more realistic model is the Gay-Berne (GB) model, informally called “anisotropic Argon”, which is still relatively simple, but has well-defined anisotropies of ellipsoidal molecular shape and intermolecular interaction. We successfully applied [2] the anisotropic GB model to revisit the theoretical grounds for the density scaling idea, which have been originally formulated mainly based on simulations in simple isotropic models such as the KABLJ mixture. It turned out that both the translational and rotational relaxation times collected at different pressures and temperatures can be scaled with the same scaling exponent γ for a given anisotropy aspect ratio, but the scaling exponent γ cannot be determined from a linear correlation between virial and potential energy, as earlier expected from simulations in isotropic models. Our findings, combined with a few others obtained earlier from MD simulations in more complex models, show the crucial role of molecular anisotropy in properly understanding the density scaling idea, the significance of which relies on the suggested relationship between macroscopic and microscopic quantities reflected in the scaling exponent γ that can be determined from measurement data, but it is also related to the exponent of the repulsive part of short-range effective intermolecular potential valid for viscous systems. 

By performing and analyzing MD simulations of a molecular liquid consisting of flexible trimers and a model polymer melt, Leporini’s group argued [3] that mutual information (MI) can be extracted on two distinct aspects of transport and relaxation near the glass transition, which are dynamic heterogeneity (DH) and secondary Johari–Goldstein (JG) relaxation processes. In the model molecular liquid with significant DH, the MI-based analysis revealed two different fractions, which were spatially grouped in clusters that exhibited different mobility and relaxation properties. However, in the case of the model polymer melt, the MI-based examinations led to the conclusion that the mechanistic explanation of both DH and relaxation must involve the rotational–translational coupling. The latter finding makes an important contribution to the long-standing discussion on the dynamic heterogeneity role in molecular dynamics of complex systems approaching the glass transition as well as our understanding of the primary and secondary relaxations and the coupling of relaxation processes. Going further in the second paper [4], Leporini’s group introduced us to the modern era of artificial intelligence (AI) applications in a simulation study of disordered condensed matter systems. The authors first performed MD simulations of a three-dimensional polymer melt of fully-flexible (i.e., without bond–bond bending and torsional potentials) linear pentamers with a total number of monomers *N* = 10,000, where non-bonded monomers interact via an LJ potential and bonded monomers interact via a harmonic potential. Then, they used the simulation data to train two slightly different neural networks (NN) and to verify the glass former molecular mobility properties predicted by the NNs in a wide range of MD timescale from fast vibrational dynamics to slow structural relaxation. As a result, they found that accurate predictions were made by this NN system, the machine learning of which included the information provided by fast vibrational dynamics and was quantified by the local Debye–Waller factor. Interestingly, both the NN systems exhibited quite impressive and rather comparable performance to predict the four-point susceptibility and primary relaxation time. It is worth noting that the role of such combined classical and AI-based methodologies in research is expected to increase significantly in the near future. 

This Special Issue includes a communication [5] paper and a review [6] devoted to new experimental ideas as well as regular articles [7,8,9] that report the specific molecular properties of materials near the glass transition, which enhance our understanding of molecular mechanisms that may result in contemporary pharmaceutical or other applications. After many years of tests and challenging considerations completed due to the team leader’s great research experience in the fields of dielectric and infrared spectroscopies, Kremer et al. [5] showed that it is possible to usefully combine those important measurement techniques. Although this novel experimental approach has some limitations that require overcoming before its inclusion in a standard set of measurement methods, it should be stressed that it provides new prospects for molecular spectroscopic techniques. This is because broadband dielectric spectroscopy (BDS) has been and remains a powerful measurement method for the investigation of molecular relaxation processes over a dozen decades of molecular dynamics in wide temperature and pressure ranges, but the molecular origins of dielectric relaxations are still a matter of debate, especially in the case of the so-called secondary relaxation processes. On the other hand, infrared (IR) spectroscopy can help us to gain insight into the molecular mechanisms, and the IR spectral range is known to have a “fingerprint” character for the intra- and intermolecular bonds of a molecule under study [5]. Thus, Kremer’s idea gives us a chance to properly relate macroscopic and microscopy phenomena near the glass transition. 

A similar goal guided Vogel and Rössler’s team members, who focused on nuclear magnetic resonance (NMR) relaxometry [6] as a source of knowledge about microscopic phenomena, which can elucidate molecular mechanisms underlying relaxation processes explored by using the dielectric or light scattering (LS) spectroscopies. The authors presented a comprehensive study of macroscopic relaxation processes as translational and rotational ones, including secondary relaxation processes and so-called “excess wing”, from the viewpoint of the NMR relaxometry applied to different material groups such as simple liquids but also including hydrogen bonds, ionic liquids, salt solutions, monohydroxy alcohols, and liquids in confinements. In the measurements, the Field-Cycling (FC) NMR technique was used as a versatile tool to determine the dynamic susceptibilities of glass-forming liquids over a broad frequency range [6]. In general, the assessment of the NMR relaxometry’s usefulness is very promising, because it allows studying translational and rotational motions and evaluating single-particle or multi-particle correlation functions. Furthermore, it can probe individual components of examined compositions. By comparing results obtained from the FC NMR reflectometry and other spectroscopic techniques such as BDS and LS, Vogel and Rössler’s team claimed, among other things, that the hotly debated excess wing considered on the high-frequency flank of the primary relaxation process is a generic relaxation feature of liquids approaching the glass transition, although its amplitude differs depending on experimental methods, which exhibit different sensitivities to small-amplitude motions. This is an important contribution to the long-standing discussion about the molecular origin of relaxation processes. 

Musioł’s team performed a detailed investigation of Schiff bases near the glass transition at ambient and elevated pressure in cooperation with a few other groups [7]. These compounds are of great interest mainly due to their various valuable applications in chemical syntheses, medicine, biotechnology, photovoltaics, electronics, and optoelectronics. However, these materials are characterized by complex patterns of relaxation behaviors that vary depending on the compounds, although they have been classified under the same class of chemical compounds. The authors focused on two Schiff bases that belong to the subgroup of glycine imino esters and differ only in one substituent, but the small structural difference resulted in large discrepancies in their physicochemical properties. By performing a series of various experimental and computational investigations based on the BDS in ambient and high-pressure conditions, differential scanning calorimetry (DSC), X-ray diffraction (XRD), Fourier transform IR spectroscopy, and density functional theory computations (DFT), it was possible to discover a few regularities in the relaxation behavior of Shiff bases in the supercooled liquid state. Among other things, the authors showed that the high thermal stability and very good glass-forming ability of Schiff bases are reflected in atypically high and tunable values of the glass transition temperature *T_g_* and the pressure coefficient of *T_g_* defined as *dT_g_*/*dp*, which turned out to satisfy the following useful relationships: *T_g_* is well described by a power law of molar mass, whereas *dT_g_*/*dp* linearly depends on the activation volume, which should simplify and better organize further investigations of this kind of chemical compounds. 

One of the leading trends in contemporary pharmaceutical sciences, which covers a complex study on amorphous pharmaceuticals, including their efficient formulation and long-term stability, has been also represented [8,9]. It is worth mentioning that many medical applications of pharmaceuticals require the better bioavailability of drugs, which is usually satisfied by disordered materials characterized by a higher water solubility than their crystalline counterparts. However, amorphous materials are often physically and even chemically unstable, and many research efforts are made to overcome problems such as the recrystallization of drugs from supercooled and glassy states and their chemical degradation, which are possible not only in laboratory tests but also when an amorphization procedure validated for a given drug in the laboratory context is scaled to technologies available in manufacturing. Macovez and Tamarit’s team members [8] thoroughly investigated drug–biopolymer dispersions to achieve a better understanding of the molecular mechanisms that underlie the physical stability of the anxiolytic drug nordazepam mixed with two similar biodegradable biopolymers, which are the homopolymer poly-L-lactide (PLLA) that is typically semicrystalline and the racemic poly-(D,L)-lactide (PDLLA) that is amorphous. The authors examined the compositions prepared in the forms of solvent-cast films and electrospun microfibers at various concentrations of components by exploiting several experimental techniques such as BDS, DSC, thermogravimetry (TGA), and scanning electron microscopy (SEM). Their comparative studies showed that biopolymers may exert both plasticizing and antiplasticizing effects on the drug–biopolymer compositions depending on the preparation method, temperature, and polymer enantiomerism, which well illustrates the mentioned challenges issued to researchers who work on the development of stable disordered solid pharmaceutical compositions. An interesting observation made by the authors relates to the secondary JG-relaxation that has been identified in even fully amorphous homogeneous mixtures in the form of two distinct JG-processes attributed separately to each component, although only a single structural relaxation was detected in most mixtures. This finding may have important implications for the interpretation of the JG-process, which is usually considered a precursor of structural relaxation, and the role of this secondary relaxation in the physical stability of the microfibers consisting of small-molecule drugs and polymers [8]. 

Within the scope of amorphous pharmaceuticals, we reported on the physical instability of the anti-inflammatory drug etoricoxib [9], which was earlier unexpected, at least in atmospheric conditions. All previous BDS and DSC investigations suggested a very high resistance of the drug to recrystallization from both the supercooled liquid and glassy states at ambient pressure. However, our systematic non-isothermal and isothermal tests for the drug recrystallization, which were performed successfully for the first time by the refractometry technique, give evidence for its recrystallization from the supercooled liquid state in isothermal conditions at ambient pressure [9]. It turned out that a different geometry and larger size of the sample in refractometric measurements compared to those employed in the standard DSC and BDS experiments may result in the physical instability of the examined drug, which exemplifies the mentioned risks possible during the manufacturing of amorphous drugs. Furthermore, we implemented a different stabilization strategy than those in [8], which is based on mixing the small-molecule drug with a small-molecule excipient octaacetylmaltose instead of macromolecule additives. Based on the performed BDS, DSC, and refractometry measurements as well as earlier investigations, we proposed a plausible molecular mechanism of the obtained satisfactory physical stability of the amorphous composition, providing a promising outcome for pharmaceutical applications in this case. 

The variety and importance of the topics addressed within the Special Issue demonstrate its necessity. Although many issues remain unsolved in the field of the glass transition and related phenomena, this Special Issue provides new ideas and perspectives for their solutions. It is my great privilege to express sincere thanks to each of the authors for their contributions, without whom this Special Issue would have not been possible.
